# Chest Compression Synchronized Ventilation versus Intermitted Positive Pressure Ventilation during Cardiopulmonary Resuscitation in a Pig Model

**DOI:** 10.1371/journal.pone.0127759

**Published:** 2015-05-26

**Authors:** Clemens Kill, Monika Galbas, Christian Neuhaus, Oliver Hahn, Pascal Wallot, Karl Kesper, Hinnerk Wulf, Wolfgang Dersch

**Affiliations:** 1 Department of Anesthesiology and Critical Care, Philipps-University, Marburg, Germany; 2 Department of Emergency Medicine, Philipps-University, Marburg, Germany; 3 Department of Internal Medicine, Section Respiratory Diseases, Philipps-University, Marburg, Germany; 4 Weinmann Emergency Medical Technology GmbH+Co.KG, Hamburg, Germany; Azienda Ospedaliero-Universitaria Careggi, ITALY

## Abstract

**Background:**

Guidelines recommend mechanical ventilation with Intermitted Positive Pressure Ventilation (IPPV) during resuscitation. The influence of the novel ventilator mode Chest Compression Synchronized Ventilation (CCSV) on gas exchange and arterial blood pressure compared with IPPV was investigated in a pig model.

**Methods:**

In 12 pigs (general anaesthesia/intubation) ventricular fibrillation was induced and continuous chest compressions were started after 3min. Pigs were mechanically ventilated in a cross-over setting with 5 ventilation periods of 4min each: Ventilation modes were during the first and last period IPPV (100% O_2_, tidalvolumes = 7ml/kgKG, respiratoryrate = 10/min), during the 2nd, 3rd and 4th period CCSV (100% O_2_), a pressure-controlled and with each chest compression synchronized breathing pattern with three different presets in randomized order. Presets: CCSV_A_: P_insp_ = 60mbar, inspiratorytime = 205ms; CCSV_B_: P_insp_ = 60mbar, inspiratorytime = 265ms; CCSV_C_: P_insp_ = 45mbar, inspiratorytime = 265ms. Blood gas samples were drawn for each period, mean arterial (MAP) and centralvenous (CVP) blood pressures were continuously recorded. Results as median (25%/75%percentiles).

**Results:**

Ventilation with each CCSV mode resulted in higher PaO_2_ than IPPV: PaO_2_: IPPV_first_: 19.6(13.9/36.2)kPa, IPPV_last_: 22.7(5.4/36.9)kPa (p = 0.77 vs IPPV_first_), CCSV_A_: 48.9(29.0/58.2)kPa (p = 0.028 vs IPPV_first_, p = 0.0001 vs IPPV_last_), CCSV_B_: 54.0 (43.8/64.1) (p = 0.001 vs IPPV_first_, p = 0.0001 vs IPPV_last_), CCSV_C_: 46.0 (20.2/58.4) (p = 0.006 vs IPPV_first_, p = 0.0001 vs IPPV_last_). Both the MAP and the difference MAP-CVP did not decrease during twelve minutes CPR with all three presets of CCSV and were higher than the pressures of the last IPPV period.

**Conclusions:**

All patterns of CCSV lead to a higher PaO_2_ and avoid an arterial blood pressure drop during resuscitation compared to IPPV in this pig model of cardiac arrest.

## Introduction

The major goal of resuscitation is producing best perfusion of vital organs to prevent tissue damage and to restore spontaneous circulation. For many years chest compressions are generally recommended to produce a blood flow through the heart and lungs when cardiac arrest appears. In addition to chest compressions there must be any supply of oxygen into the lungs to avoid desaturation of the arterial blood [1]. While in the first minutes of cardiac arrest active ventilation of the lungs seems to be of minor importance and might be not necessary at all [2–4], Intermitted Positive Pressure Ventilation is generally recommended once Advanced Life Support (ALS) is started [5–7]. Nevertheless there exist only limited data on the effects of positive pressure ventilation on gas exchange as well as on haemodynamics during cardiopulmonary resuscitation [8–10].

While the inventors of chest compressions believed in the effects of direct compressions to the heart to produce a blood flow, we meanwhile know that sudden changes in intrathoracic pressure also cause a blood flow through the heart and lungs. More than forty years ago Criley et al. described a phenomen named “cough-resuscitation”, where strong and sudden changes of the airway pressure caused by coughing in the beginning of cardiac arrest were able to produce a blood flow without any chest compression at all. By this procedure the patient could be kept awake for one or two minutes during ventricular fibrillation until defibrillation and standard ALS including chest compressions was started [11].

Out of these observations we developed a novel ventilator mode called Chest Compression Synchronized Ventilation (CCSV), that is designed to insufflate a short positive pressure ventilation exactly in time with the start of each chest compression and was recently published [12]. This principle might be described as an “artificial cough resuscitation effort” added on each chest compression. In this study we investigated the influence of Intermittent Positive Pressure Ventilation (IPPV) in volume-controlled mode compared to three different presets of Chest Compression Synchronized Ventilation (CCSV) in a cross-over design in a pig model of cardiac arrest to evaluate possible effects of different CCSV pressure-time curves. Primary endpoint was the arterial oxygenation, secondary endpoints were the decarboxylation, acid base state and the arterial pressure depending on the ventilation mode.

## Methods

### Animal preparation

In accordance with German animal protection law (TierSchG v.18. Mai 2006 BGBl. I S. 1206, 1313), the protocol was approved by institutional animal protection commissioner and by the local governmental authority (Regierungspraesidium Giessen, V54-19c20-15(1) MR20/13Nr76/2010). The study was performed on a total of twelve pigs (Sus scrofa domestica, Deutsche Landrasse) using a pig model as described before [12,13].

The animals were premedicated with intramuscular application of 20 mg∙kg^-1^ ketamine, 0.03 mg∙kg^-1^ atropine and 1 mg∙kg^-1^ diazepam. An IV-line was inserted into an ear vein and after induction of anaesthesia with i.v. 1μg∙kg^-1^ sufentanil and 3mg∙kg^-1^ propofol, endotracheal intubation was performed (ID 6.0 mm). Anaesthesia was maintained with propofol infusion (2–3mg∙kg^-1^∙h^-1^). The right femoral artery, right femoral vein and right internal jugular vein were fitted with catheters for invasive blood pressure transducers and probe sampling. Haemodynamic and ventilation parameters were recorded continuously. The pigs were placed and adjusted in the pneumatic driven Lund University Cardiac Arrest System LUCAS 1 (Medtronic GmbH, Germany) in the supine position with deactivation of the active-decompression-mode by a coverage sheet between the stamp of the device and the sternum. To avoid dislocation and uncontrolled movement during chest-compression, an adjustable U-shaped fixing frame was used. During a steady-state period before inducing cardiac arrest, ventilation was performed with IPPV and room air and calibrated to an arterial PaCO_2_ of 5.3kPA(40mmHg). To avoid gasping during resuscitation, i.v. rocuronium (1mg*kg^-1^) was administered before inducing cardiac arrest.

### Experimental protocol

Ventricular fibrillation (VF) was induced with a right ventricular paced electrode and AC 7.5 to 15V. VF remained untreated for 3 minutes without any ventilation or chest compression. With the beginning of chest compression a t = 3min, mechanical ventilation was performed for five periods of four minutes each. Mechanical ventilation was applied with the emergency and transport ventilator MEDUMAT Transport (Weinmann GmbH+Co.KG, Germany).

During the first and last period ventilation was applied with IPPV at a volume-controlled mode with a constant flow without PEEP, FiO_2_ 1.0, fixed tidal volume of 7ml/kg, respiratory rate 10/min and I:E 1:1.5 (resulting in an inspiratory time of 2.4 s and expiratory time 3.6 s), the upper airway pressure limit was set to 60 mbar.

**Fig 1:**
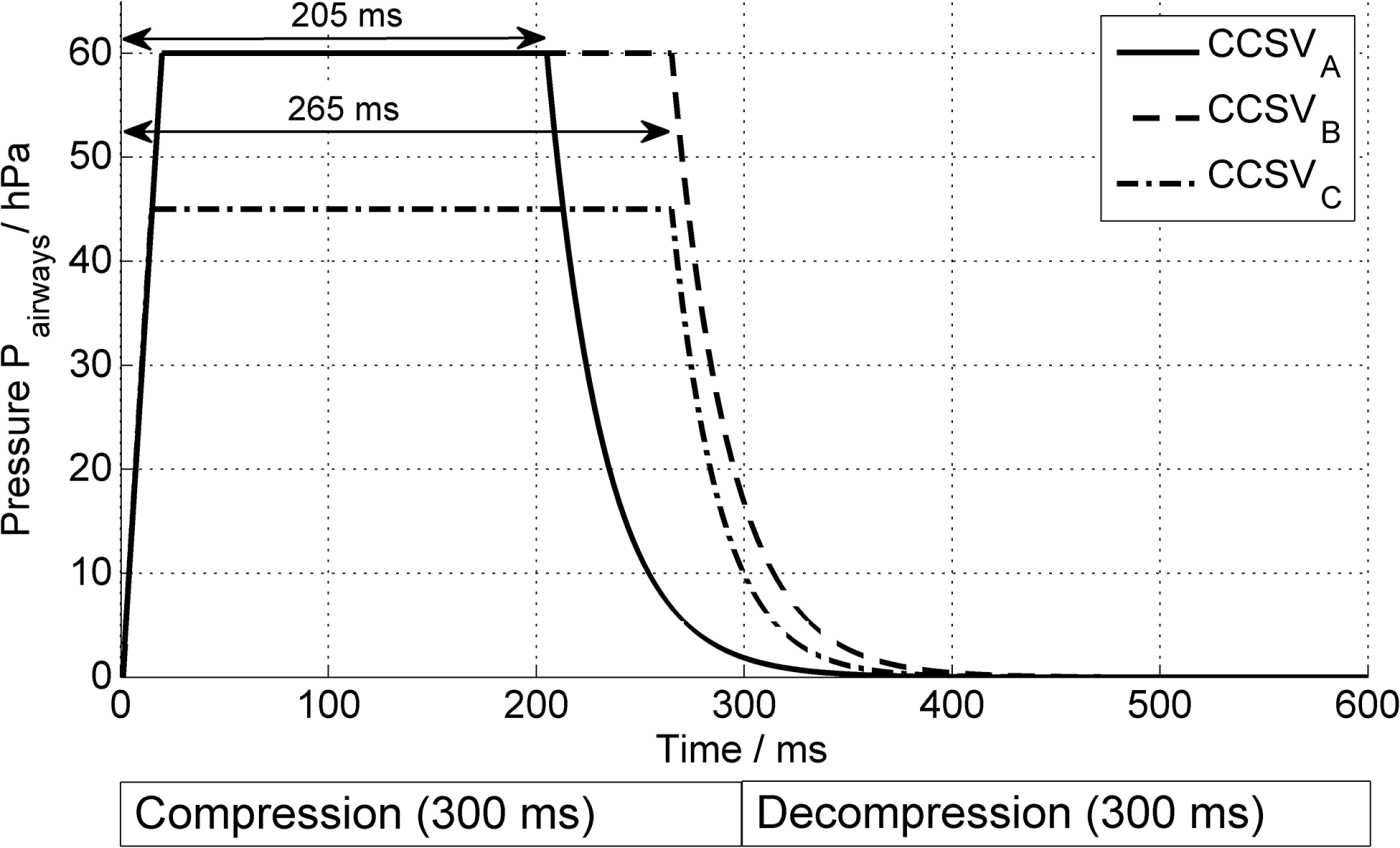
Pressure-time-curves of CCSV. Pressure-time-curves of the three presets of Chest Compression Synchronized ventilation CCSV_A_, CCSV_B_, and CCSV_C_ depending on compression-decompression-cycle.

For the 2nd, 3rd and 4th ventilation period the novel CCSV was implemented by reprogramming the Pressure Support Ventilation mode comprising an inverse trigger, cycling mechanisms and higher inspiratory pressure levels up to 60 mbar. The goal of the new inverse trigger is to detect starting chest compression efforts and to initiate an instant inspiratory pressure. Different trigger levels can be set by the user to adjust to different resuscitation situations (e.g., due to different chest compression patterns and patient chest and lung mechanics). The new trigger is activated by reaching three criteria at the same time: The first criterion is achieved when the airway pressure rises above a certain trigger level of 0.9 to 3.7 mbar above PEEP. The second criterion is when the airways pressure gradient reaches at least 25 to 375 mbar/s (i.e., when pressure rises fast enough) and the last criterion is met when at least 200 to 340ms of expiration (chest decompression) have occurred. In summary, the new trigger is true when the airway pressure rises fast enough above a certain pressure after a minimal time of expiration. The cycling (switching back to expiration) of a CCSV inspiration can be adjusted to different fixed inspiration times between 205 and 340 ms. For this study the different presets of the CCSV periods with 100% O_2_ were: CCSV_A_: P_insp_ = 60mbar, inspiratory time 205ms; CCSV_B_: P_insp_ = 60mbar, inspiratory time 265ms; CCSV_C_: P_insp_ = 45mbar, inspiratory time 265ms. PEEP was set to 0 mbar to avoid incomplete expiration and to allow unhampered venous blood flow into the right heart. Fig 1 describes the pressure-time curves of the different CCSV presets.

**Fig 2:**
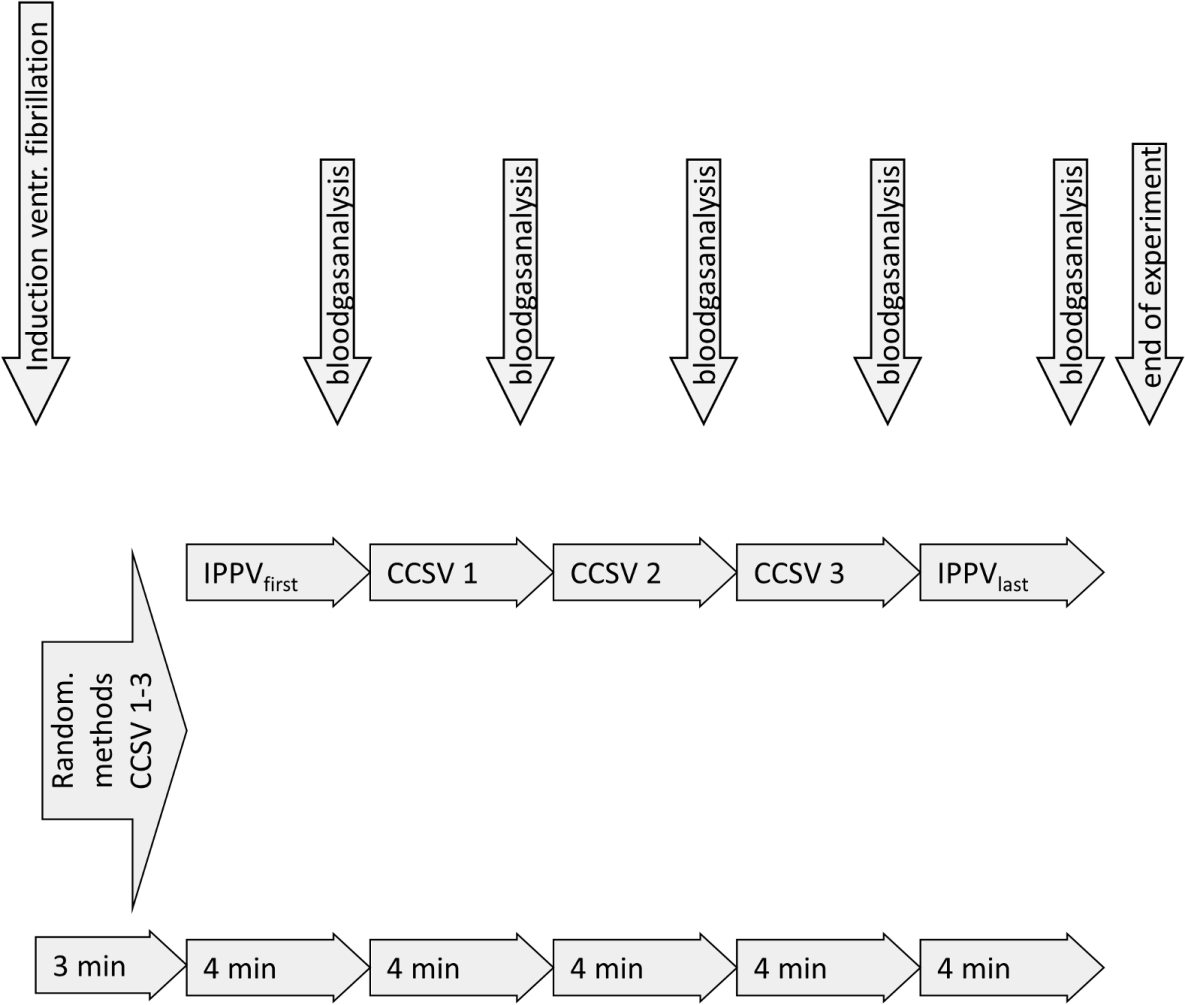
Timeline of experimental procedure. Timeline of induction of cardiac arrest followed by five ventilation periods using IPPV and CCSV.

The three presets of CCSV for the 2nd, 3rd and 4th ventilation period were applied in a randomized order using a sealed envelope randomization to reduce the influence of total duration of CPR on the effects on gas exchange and haemodynamics. Based on the inclusion of 12 pigs each of the three CCSV presets was thereby allocated twice on each ventilation period, while both IPPV periods were allocated as the first and the last (5th) ventilation period in all experiments as control group. Fig 2 shows the time line of the experimental procedure.

Arterial and mixed venous blood gas samples were drawn at t = 0min, t = 7min, t = 11min, t = 15min, t = 19min and t = 23 minutes. Arterial and central venous pressures (CVP) were recorded continuously with a sampling rate of 100Hz. Mean arterial pressure (MAP) and the difference of arterial pressure and central venous pressure (MAP-CVP) were calculated for each intervention period.

At the end of the experiment the animals were sacrificed by infusion of potassiumchloride. During surgery and all experimental procedures analgesia and anaesthesia were maintained without regaining consciousness. Post mortem all animals were investigated to detect pneumothorax by chest sonography.

### Statistical methods

Due to the small sample size and therefore anticipated violation of Gaussian distribution of values, parametric tests were not considered adequate. Instead, non-parametric tests were applied. The Type-I error rate was defined at alpha = 0.05. Descriptives used to describe data were also non-parametric, including the median, inter-quartile distances as well as minimum and maximum. For a simultaneous comparison with regard to central tendency for all treatment modalities (IPPV_first_, CCSV_A_, CCSV_B_, CCSV_C_, IPPV_last_) applied in a cross-over design, the Friedman-Test with multiple post-hoc comparisons was performed. For assessing statistical significance of single comparisons, p-values with Bonferroni-Holm correction were used. This was done for all primary and secondary outcome variables.

### Sample size determination

The randomized allocation oft the three intervention groups CCSV_A_, CCSV_B_, and CCSV_C_ on three possible ventilation periods per time resulted in the following six possible allocation orders:

IPPV_first_-CCSV_A_-CCSV_B_-CCSV_C_-IPPV_last_

IPPV_first_-CCSV_A_-CCSV_C_-CCSV_B_-IPPV_last_

IPPV_first_-CCSV_B_-CCSV_A_-CCSV_C_-IPPV_last_

IPPV_first_-CCSV_B_-CCSV_C_-CCSV_A_-IPPV_last_

IPPV_first_-CCSV_C_-CCSV_A_-CCSV_B_-IPPV_last_

IPPV_first_-CCSV_C_-CCSV_B_-CCSV_A_-IPPV_last_

Therefore the total number of experiments had o be 6 or a multiple of 6. Based on a study design for repeated measures, an alpha of 0.05, a power of 80% and an estimated effect size of 0.9 (primary outcome parameter PaO_2_, calculation based on previous data^12^) resulted in a sample size of n = 12.

### Powercalculation

Based on a study design for repeated measures, an alpha of 0.05, a sample size of n = 12 and an estimated effect size of 0.9 (primary outcome parameter PaO_2_, calculation based on previous data [12]) resulted in a power of 77%.

All statistical calculations were performed using the statistical software packages SPSS (Version 22) and BiAS for Windows (Version 10.04, Epsilon-Verlag, 2013).

## Results

Data from all 12 animals (median weight 51.75 (50.9/56.1)kg) were included in the analysis. The baseline values of arterial blood gas analysis during ventilation with room air immediately before induction of cardiac arrest were in median (25/75% percentiles) PaO_2_ 9.6 (8.6/10.4) kPa, PaCO_2_ 5.3 (5.0/5.5) kPa and pH 7.48 (7.48/7.51).

**Fig 3:**
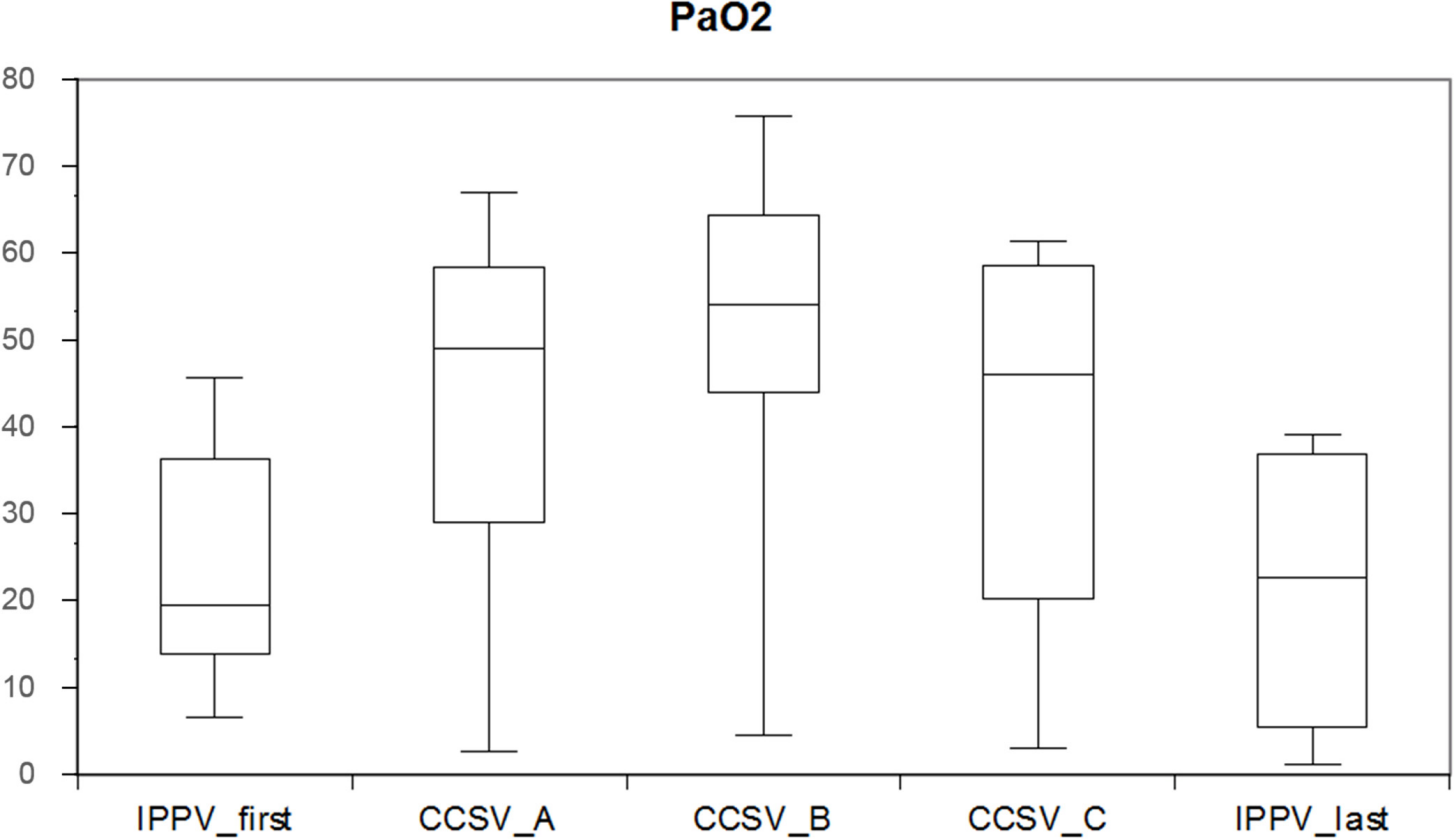
Results of PaO_2_. Results of PaO_2_ of IPPV_first_, CCSV_A_, CCSV_B_, CCSV_C_, and IPPV_last_ (median, 25/75% percentiles, min/max) [kPa].

The primary endpoint arterial oxygenation showed significantly higher PaO_2_ in all CCSV modes with more than double of the values of both IPPV periods. There were no significant differences in PaCO_2_, but both CCSV_c_ (inspiratory peak pressure 45mbar) and IPPV _last_ resulted in a median PaCO_2_ above 7 kPa (Table 1 and Fig 3).

**Table 1:** Blood gas results.

Group(n = 12)	Blood gas parameterMedian (25/75percent)	P-values between groups			
	arterial pH	CCSV_A_	CCSV_B_	CCSV_C_	IPPV_last_
IPPV_first_	7.39 (7.33/7.45)	0.38	1	0.06	0.02
CCSV_A_	7.27 (7.21/7.37)	—	0.43	1	0.78
CCSV_B_	7.33 (7.28/7.44)		—	0.08	0.03
CCSV_C_	7.25 (7.19/7.34)			—	1
IPPV_last_	7.23 (7.1/7.41)				—
	PaO_2_ [kPa]	CCSV_A_	CCSV_B_	CCSV_C_	IPPV_last_
IPPV_first_	19.6 (13.9/36.2)	0.028	0.001	0.006	0.77
CCSV_A_	48.9 (29.0/58.2)	—	0.77	1	0.0001
CCSV_B_	54.0 (43.8/64.1)		—	1	0.0001
CCSV_C_	46.0 (20.2/58.4)			—	0.0001
IPPV_last_	22.7 (5.4/36.9)				—
	PaCO_2_ [kPa]	CCSV_A_	CCSV_B_	CCSV_C_	IPPV_last_
IPPV_first_	6.5 (5.5/7.0)	n.s.	n.s.	n.s.	n.s.
CCSV_A_	6.2 (5.2/7.8)	—	n.s.	n.s.	n.s.
CCSV_B_	6.1 (4.2/7.3)		—	n.s.	n.s.
CCSV_C_	7.8 (6.3/8.8)			—	n.s.
IPPV_last_	7.7 (4.2/10.3)				—
	venous pH	CCSV_A_	CCSV_B_	CCSV_C_	IPPV_last_
IPPV_first_	7.32 (7.28/7.36)	0.022	0.009	0.022	<0.0001
CCSV_A_	7.24 (7.2/7.3)	—	1	1	0.0004
CCSV_B_	7.26 (7.23/7.3)		—	1	0.001
CCSV_C_	7.26 (7.2/7.3)			—	0.0004
IPPV_last_	7.19 (7.15/7.25)				—
	PvO_2_ [kPa]	CCSV_A_	CCSV_B_	CCSV_C_	IPPV_last_
IPPV_first_	3.5 (2.9/4.0)	n.s.	n.s.	n.s.	n.s.
CCSV_A_	3.3 (3.1/3.8)	—	n.s.	n.s.	n.s.
CCSV_B_	3.3 (3.2/3.4)		—	n.s.	n.s.
CCSV_C_	3.0 (2.7/3.6)			—	n.s.
IPPV_last_	3.3(2.8/3.5)				—
	PvCO_2_ [kPa]	CCSV_A_	CCSV_B_	CCSV_C_	IPPV_last_
IPPV_first_	8.0 (7.4/8.3)	0.167	0.052	0.061	<0.0001
CCSV_A_	8.6 (7.7/9.9)	—	1	1	0.052
CCSV_B_	8.4 (8.1/9.6)		—	1	0.167
CCSV_C_	8.4 (7.7/10.5)			—	0.139
IPPV_last_	9.2 (8.0/10.6)				—
	SvO_2_ [%]	CCSV_A_	CCSV_B_	CCSV_C_	IPPV_last_
IPPV_first_	39.5 (32/50)	1	1	0.048	0.0041
CCSV_A_	34.5 (31.3/38)	—	1	0.337	0.051
CCSV_B_	34.5 (34/36.8)		—	0.051	0.0046
CCSV_C_	32 (24.5/35.8)			—	1
IPPV_last_	28.5 (24.3/31)				—

Arterial and mixed venous blood gas results.

**Fig 4:**
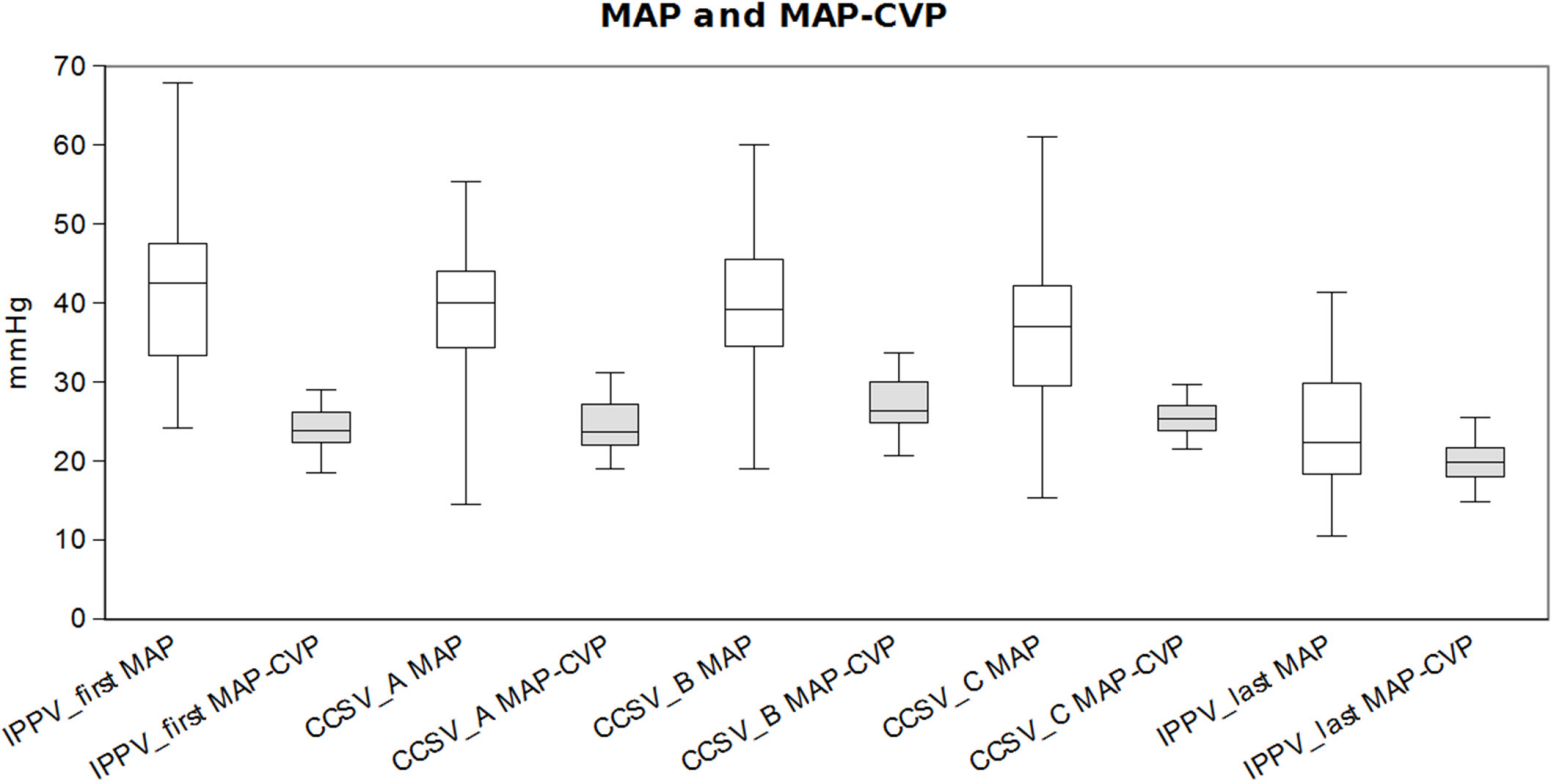
MAP and MAP-CVP. Results of mean arterial pressure (MAP) and difference of mean arterial pressure and central venous pressure (MAP-CVP) (median, 25/75% percentiles, min/max) [mmHg].

The mean arterial pressures were comparable to IPPV_first_ for all CCSV modes and significantly higher to IPPV_last_. The difference MAP-CVP was similar (CCSV_A_) or slightly elevated (CCSV_B_, CCSV_C_) compared to IPPV_first_ and all CCSV modes were significantly higher than IPPV_last_. (Table 2 and Fig 4).

**Table 2:** Results of MAP and MAP-CVP.

Group(n = 12)	Blood pressuresMedian (25/75percent)	P-values between groups			
	MAP [mmHg]	CCSV_A_	CCSV_B_	CCSV_C_	IPPV_last_
IPPV_first_	42.5 (33.4/47.5)	1	1	0.95	<0.0001
CCSV_A_	40.1 (34.4/44)	—	1	1	<0.0001
CCSV_B_	39.2 (34.5/45.6)		—	1	<0.0001
CCSV_C_	37 (29.5/42.2)			—	<0.0001
IPPV_last_	22.4 (18.4/29.9)				—
	MAP-CVP [mmHg]	CCSV_A_	CCSV_B_	CCSV_C_	IPPV_last_
IPPV_first_	24 (22.3/26.2)	0.37	<0.0001	<0.0001	<0.0001
CCSV_A_	23.7 (22.1/27.2)	—	<0.0001	0.0011	<0.0001
CCSV_B_	26.5 (24.9/30)		—	<0.0001	<0.0001
CCSV_C_	25.4 (23.9/27)			—	<0.0001
IPPV_last_	19.9 (18/21.7)				—

Results of mean arterial pressure (MAP) and difference of mean arterial pressure and central venous pressure (MAP-CVP).

**Fig 5:**
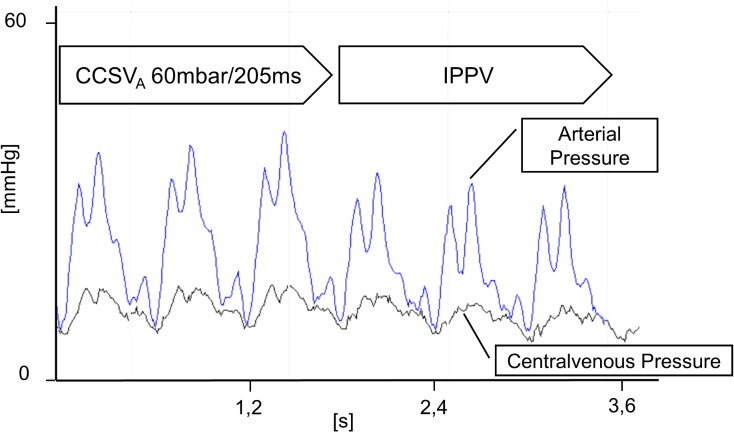
Arterial and centralvenous pressure curves. Arterial and centralvenous pressure curves of experiment No. 2 at t = 19min showing the change of ventilation mode from CCSV_A_ (with an inspiratory peak pressure of 60mbar and inspiratory time of 205ms) to IPPV mode.

The change of ventilation mode from CCSV to IPPV_last_ lead to a typical drop in arterial blood pressure as shown in Fig 5.

The measured inspiratory peak pressures were during both IPPV ventilation periods below the upper pressure limit of 60mbar, the maximum measured peak pressure was 33 mbar and no break-off of any IPPV tidalvolume could be observed.

The measured inspiratory peak pressures during the pressure controlled periods of CCSV were in median (25/75% percentiles): CCSV_A_ 57 (56/ 57)mbar and CCSV_B_ 61 (61/62)mbar; CCSV_C_ 45 (44/45)mbar.

None of the animals showed pneumothorax.

## Discussion

This study compared mechanical ventilation by an automated transport ventilator using Intermittent Positive Pressure Ventilation (IPPV) and a novel ventilator mode for resuscitation called Chest Compression Synchronized Ventilation (CCSV). CCSV led to superior arterial oxygen partial pressures and similar arterial carbon dioxide values. During a total time of twenty minutes of chest compressions without vasoconstrictors, ventilation with CCSV kept the arterial blood pressure constant, whereas IPPV lead to lower arterial blood pressures.

The Guidelines of CPR recommend the use of an automated transport ventilator with tidal volumes of 6 to 7ml/kg and a frequency of 10 per minute with pure oxygen to avoid uncontrolled ventilation once the airway is secured [6,14,15]. Although mechanical ventilation is quite common during resuscitation in and out of hospital, there are only a few investigations about the best way of applying mechanical ventilation during CPR [8,16,17]. In 2010, Yannopoulos et al. demonstrated that positive pressure ventilation improved outcome compared to chest compression only without any ventilation at all in a model of prolonged cardiac arrest [18].

Nevertheless there are major concerns about possible negative effects of positive pressure ventilation during CPR, because an increased pressure during inspiration might compromise venous blood flow into the right heart during the decompression period [19,20]. These effects might reduce the cardiac output during resuscitation with chest compressions. Based on these considerations it seems to be of great importance when exactly the raise of pressure for inspiration will be timed [21].

There are contradicting data about the influence of hyperventilation on haemodynamics. Aufderheide et al. found that reduced PaCO_2_ leads to hypotension [14,15], whereas the group of Gazmuri recently could demonstrate, that even a markedly increased minute-volume of ventilation did not negatively affect perfusion pressures [16]. On the other hand cardiopulmonary blood flow can be increased by using an impedance threshold device (ITD) alone or together with active decompression CPR [22–26]. These both strategies enhance blood flow by decreasing the intrathoracic pressure during decompression phase in order to enhance the venous blood flow into the right heart between each single compression.

The novel CCSV mode was designed to secure oxygenation and decarboxylation without negative effects on blood flow and arterial blood pressure as described above. Therefore oxygen is insufflated only simultaneously with the start of each chest compressions and these very short positive pressure ventilations are stopped before the decompression period begins to allow unhampered venous blood flow into the right heart. This simultaneous positive pressure ventilation exactly in time with the beginning of each chest compression prevents a loss of intrathoracic pressure via the airway and squeezes out the pulmonary vessels, which might enhance the cardio-pulmonary blood flow. These effects are similar to the principle of vest-CPR with compression of the whole chest as it can be performed with a load-distribution device [27,28]. Reflecting the results of arterial blood gas analysis it is interesting, that the PaO_2_ is relatively low during both IPPV periods although ventilation was performed with pure oxygen and without any pre-existing pulmonary dysfunction of the animals. This might be a sign for lower pulmonary perfusion compared to periods of ventilation with CCSV as well as for a serious ventilation/perfusion mismatch during the combination of chest compression and ventilation with IPPV.

The three different presets of CCSV were equivalent in superior arterial oxygenation, the preset CCSV_c_ with a peak inspiratory pressure of 45mbar instead of 60mbar-and therefore lower tidal volumes- was associated with the highest PaCO_2_ and a lower mixed venous oxygen saturation than CCSV_A_ and CCSV_B_.

The CCSV presets CCSV_B_ and CCSV_C_ with the longest inspiration time of 265ms were associated with the highest values for the pressure difference between MAP and CVP as the effective perfusion pressure.

Bringing these results together, a CCSV preset with an inspiratory peak pressure of 60 mbar and an inspiration time of up to 265 ms seems to be the most effective method, when chest compressions were performed with a rate of 100/min.

### Limitations

The goal of this study was to evaluate differences between volume-controlled, asynchronous Intermitted Positive Pressure Ventilation and several ventilator settings of Chest Compression Synchronized Ventilation with regard to gas exchange and blood pressure. These results are limited by the cross-over design of our study, that could not detect differences in outcome of resuscitation efforts. A second limitation are the relatively short periods of only four minutes for each ventilation mode, that were chosen because of the expeditious worsening of artificial circulation during prolonged CPR. These short periods of only four minutes each might have prevented more obvious differences between the groups mainly in the lab results, although the total duration of CPR of twenty minutes might explain the differences of the first and last IPPV ventilation period.

According to the cross-over design with application of all modes in each animal we did not perform lung histology in this study to detect pulmonary damage, that might be caused by the relatively high peak inspiratory pressures of the CCSV modes. A further limitation is the unknown pressure in the lower airway as well as the transpulmonary pressure itself during chest compression without and with different ventilation modes, that could be helpful in estimating possible adverse effects on the lung tissue.

## Conclusion

In this pig model of cardiac arrest mechanical ventilation with Chest Compression Synchronized Ventilation resulted in improved arterial oxygenation and better maintenance of arterial blood pressure than ventilation with volume controlled non-synchronized Intermitted Positive Pressure Ventilation. Further investigations should be undertaken to evaluate both the safety as the effectiveness of the ventilator mode CCSV on outcome in human cardiac arrest.

## Supporting Information

S1 Dataset (XLS)

S1 ARRIVE Checklist (PDF)
